# Identification and classification of brain tumor MRI images with feature extraction using DWT and probabilistic neural network

**DOI:** 10.1007/s40708-017-0075-5

**Published:** 2018-01-08

**Authors:** N. Varuna Shree, T. N. R. Kumar

**Affiliations:** Department of CS&E, MSRIT, Bangalore, India

**Keywords:** Image segmentation, MRI, DWT, Morphology, GLCM, PNN

## Abstract

The identification, segmentation and detection of infecting area in brain tumor MRI images are a tedious and time-consuming task. The different anatomy structure of human body can be visualized by an image processing concepts. It is very difficult to have vision about the abnormal structures of human brain using simple imaging techniques. Magnetic resonance imaging technique distinguishes and clarifies the neural architecture of human brain. MRI technique contains many imaging modalities that scans and capture the internal structure of human brain. In this study, we have concentrated on noise removal technique, extraction of gray-level co-occurrence matrix (GLCM) features, DWT-based brain tumor region growing segmentation to reduce the complexity and improve the performance. This was followed by morphological filtering which removes the noise that can be formed after segmentation. The probabilistic neural network classifier was used to train and test the performance accuracy in the detection of tumor location in brain MRI images. The experimental results achieved nearly 100% accuracy in identifying normal and abnormal tissues from brain MR images demonstrating the effectiveness of the proposed technique.

## Introduction

In image processing, images convey the information where input image is processed to get output also an image. In today’s world, the images used are in digital format. In recent times, the introduction of information technology and e-healthcare system in medical field helps clinical experts to provide better health care for patients. This study reveals the problem segmentation of abnormal and normal tissues from MRI images using gray-level co-occurrence matrix (GLCM) feature extraction and probabilistic neural network (PNN) classifier. The brain tumor is an abnormal growth of uncontrolled cancerous tissues in the brain. A brain tumor can be benign and malignant. The benign tumor has uniformity structures and contains non-active cancer cells. The malignant tumor has non-uniformity structures and contains active cancer cells that spread all over parts.

According to world health organization, the grading system scales are used from grade I to grade IV. These grades classify benign and malignant tumor types. The grade I and II are low-level grade tumors while grade III and IV are high-level grade tumors. Brain tumor can affect individuals at any age. The impact on every individual may not be same. Due to such a complex structure of human brain, a diagnosis of tumor area in brain is challenging task.

The malignant-type grade III and IV of tumor is fast growing. Affects the healthy brain cells and may spread to other parts of the brain or spinal cord and is more harmful and may remain untreated. So detection of such brain tumor location, identification and classification in earlier stage is a serious issue in medical science. By enhancing the new imaging techniques, it helps the doctors to observe and track the occurrence and growth of tumor-affected regions at different stages so that they can take provide suitable diagnosis with these images scanning.

The key issue was detection of brain tumor in very early stages so that proper treatment can be adopted. Based on this information, the most suitable therapy, radiation, surgery or chemotherapy can be decided. As a result, it is evident that the chances of survival of a tumor-infected patient can be increased significantly if the tumor is detected accurately in its early stage.

The segmentation was employed to determine the affected tumor part using imaging modalities. Segmentation is process of dividing the image to its constituent parts sharing identical properties such as color, texture, contrast and boundaries.

The research paper is organized as follows: Sect. 2 presents the related works literature survey, Sect. 3 presents the materials and methods with the steps used in the proposed technique, Sect. 4 presents the results and discussion, Sect. 5 presents the performance analysis, and finally Sect. 6 contains the conclusion and future scope.

## Literature survey

Analyzing and processing of MRI brain tumor images are the most challenging and upcoming field. Magnetic resonance imaging (MRI) is an advanced medical imaging technique used to produce high-quality images of the parts contained in the human body and it is very important process for deciding the correct therapy at right stage for tumor-infected individual.

Many techniques have been proposed for classification of brain tumors in MR images such as fuzzy clustering means (FCM), support vector machine (SVM), artificial neural network (ANN), knowledge-based techniques, and expectation-maximization (EM) algorithm technique which are some of the popular techniques used for region-based segmentation and so to extract the important information from the medical imaging modalities.

Bahadure et al. proposed BWT and SVM techniques image analysis for MRI-based brain tumor detection and classification. In this method, accuracy of 95% was achieved using skull stripping which eliminated all non-brain tissues for the detection purpose [1]. Joseph et al. [2] proposed segmentation of MRI brain images using *K*-means clustering algorithm along with morphological filtering for the detection of tumor images. The automated brain tumor classification of MRI images using support vector machine was proposed by Alfonse and Salem [3]. The accuracy of a classifier was improved using fast Fourier transform for the extraction of features and minimal redundancy maximal relevance technique was used for reduction of features. The accuracy obtained from this proposed work was 98%.

The brain MRI image contains two regions which are to be separated for the extraction of brain tumor regions. One part of region contains the tumor abnormal cells, whereas the second region contains the normal brain cells [4]. For the brain tumor segmentation, Zanaty [5] proposed an approach based on hybrid type, with the combination of seed growing, FCM, and Jaccard similarity coefficient algorithm with the measure of gray and white segmented tissue matter from tumor images. An average score of S of 90% segmentation was achieved with noise level of 9–3%.

To manage and to address protocols of different images and nonlinearity of real data an effective classification based on contrast of enhanced MRI images, Yao et al. [6] proposed an methodology which included extraction of textures features with wavelet transform and SVM with an accuracy of 83%. For the classification and brain tumor segmentation, Kumar and Vijayakumar [7] proposed methodology using principal component analysis (PCA) and radial basis function kernel with SVM. They obtained an accuracy of 94% with this method. An artificial neural network tool as both classifier and segmentation was used for the effective classification of brain tumor from MRI images was proposed by Sharma et al. [8] with the utilization of textural primitive features which achieved an accuracy of 100%.

For the medical image segmentation, a localized fuzzy clustering with the extraction of spatial information was proposed by Cui et al. [9]. The author used Jaccard similarity index as a measure of segmentation claiming an accuracy of 83–95% and differentiating in to white, gray and cerebrospinal fluid.

For the brain tumor image segmentation, active contour method was applied to solve the problem based on intensity homogeneities on MRI images was proposed by Wang et al. [10]. For the automatic extraction of features and tumor detection a with an enhanced feature using Gaussian mixture model applied on MRI images with wavelet features and principal component analysis was proposed by Chaddad [11] with an accuracy of T1- weighted 95% and T2- weighted 92% for FLAIR MRI weighted images.

The author Sachdeva et al. [11] used an artificial neural network and PCA–ANN for the multiclass brain tumor MRI images classification, segmentation with dataset of 428 MRI images and an accuracy of 75–90% was achieved.

The literature survey above gives a clear view of the techniques that were invented only to obtain the segmentation—region of interest, some techniques for extracting features and some to train and test using the classifiers for classification only. Much effective segmentation with the combined feature extraction could not be conducted, and only few features were extracted which resulted in low accuracy in tumor identification and detection. The classifiers used to train the features are also not much effective.

In this paper, we have combined discrete wavelet transformation (DWT) with the extraction of textural and GLCM features followed by morphological operations with probabilistic neural network as a classifier tool. The study deals with the extraction of features from the segmented region to detect and classify the normal and abnormal tumor cells of medical brain MRI images for a large database. Our outcome leads to conclusion that with this proposed method it makes clinical experts easy to take a decision regarding diagnosis and also scanning.

## Proposed methodology

This describes the materials, the source from which the brain image data collected and the algorithms for brain MRI segmentation and feature extraction. The methodology proposed includes application on brain MRI images of 256 × 256, 512 × 512 pixel size on dataset. It is converted into gray scale for further enhancement. The following discussion deals with implementation of algorithm.

### Preprocessing

The preprocessing step improves the standard of the brain tumor MR images and makes these images suited for future processing by clinical experts or imaging modalities. It also helps in improving parameters of MR images. The parameters includes improvement in signal-to-noise ratio, enhancement in visual appearance of MR images, the removal of irrelevant noise and background of undesired parts, smoothing regions of inner part, maintaining relevant edges [12].

#### Segmentation

The segmentation is a process where the image is partitioned into different regions. Let an entire region of image be represented by S. Segmentation process can be viewed as partition of S into p subregions like S1, S2, S3, …Sp. Certain conditions has to satisfied such as the segmentation must be intact; that is each and every pixel should be within the region, every points in the regions should be connected in some sense, regions should be disjoint, etc.

#### Region growing

Region growing is grouping of pixels or subregions into larger regions based on certain criteria. The main aim was to select a ‘seed’ points and attach each of these seed to those neighboring pixels having identical properties to grow region. A set of seeds was taken as input within the image and marked the objects to be segmented. The region grows iteratively by estimating all unallocated neighboring pixels of the region. The similarity was the measure of difference between pixel’s intensity value and the region’s mean, *δ*. The pixel with the smallest difference measured this way was allocated to the respective region. This was continued until all pixels were allocated to a region. Seeded region growing requires seeds as additional input. The results depend on the selection of seeds [13]. The measurement was based on mean value of the pixel intensity. The image gets segmented; this image was used to identify the desired tumor region.

### Morphological operations

Morphology deals with study of shapes and boundary area extraction from brain tumor images. Morphological operation is rearranging the order of pixel values. It operates on structuring element and input images. Structuring elements are attributes that probes a features of interest. The basic operations used here are dilation and erosion. Dilation operation adds the pixels to boundary region, while erosion removes the pixels from the boundary region of the objects. These operations were carried out based on the structuring elements. Dilation chooses highest value by comparing all pixel values in neighborhood of input image described by structuring element, whereas erosion chooses the lowest value by comparing all the pixel values in the neighborhood of the input image [14].

### Feature extraction

Feature extraction is process of extracting quantitative information from an image such as color features, texture, shape and contrast. Here, we have used discrete wavelet transform (DWT) for extracting wavelet coefficients and gray-level co-occurrence matrix (GLCM) for statistical feature extraction.

#### Feature extraction using DWT

The wavelet was used to analyze different frequencies of an image using different scales. Here, we are using discrete wavelet transform (DWT) which is powerful tool for feature extraction. It was used to extract coefficient of wavelets from brain MR images. The wavelet localizes frequency information of signal function which was important for classification.

2D discrete wavelet transform was applied that resulted in four subbands LL(low–low), HL(high–low),LH(low–high), HH(high–high) with the two-level wavelet decomposition of Region of Interest (ROI). The 2D level decomposition of an image displays an approximation with detailed three images that represents low and high-level frequency contents in an image, respectively [15]. The wavelets approximations at first and second level are represented by LL1, LL2, respectively; these represent the low-frequency part of the images. The high-frequency part of the images are represented by LH1, HL1, HH1, LH2, HL2 and HH2 which gives the details of horizontal, vertical and diagonal directions at first and second level, respectively. We have used low-level image, where LL1 represents the approximation of original image and is further decomposed to second-level approximation and details of image. The process was repeated until we obtained the desired level of resolution.

By using 2D discrete wavelet transform, the images were decomposed into spatial frequency components were extracted from LL subbands and since HL subbands have higher performance when compared to LL, we have used both LL and HL for better analysis which describes image text features [16]. The different frequency components and each component were studied with resolution matched to its scale and expressed as:

1 $$ {\text{DWT}}\;p({\text{s}}) = \left\{ {_{{di,j\,\,=\,\,\sum {p({\text{s}})g\,*\,i({\text{s}} - 2ij)} }}^{{di,j\,\,=\,\,\sum {p({\text{s}})h\,*\,i({\text{s}} - 2ij)} }} } \right. $$

The coefficients *d*_*i*,*j*_ refers to the component attribute in signal *p*(s) corresponding to the wavelet function, whereas *b*_*i*,*j*_ refer to the approximated components in the signal. The functions h(s) and g(s) in the equation represent high-pass and low-pass filters coefficients, respectively, while parameters *i* and *j* refer to wavelet scale and translation factors.

#### Feature extraction using GLCM

Texture analysis differentiates normal and abnormal tissues easily for human visual perception and machine learning. It also provides variation between malignant and normal tissues, which may not be visible to human eye. It improves the accuracy by choosing effective quantitative features for early diagnosis. In the first step, the first-order statistical textural analysis-features information from the histogram of image intensities was extracted and frequencies of gray level at a random image positions were measured. It does not consider correlation or co-occurrences, between pixels. In the second step, the second-order textural analysis-features were extracted based on probability of gray levels at random distances and over entire image orientations.

The statistical features were extracted using gray-level co-occurrence matrix (GLCM), also known gray-level spatial dependence matrix (GLSDM). GLCM was introduced by Haralick [17]. It is an approach that describes the spatial relation between pixels of various gray-level values [15]. Gray-level co-occurrence matrix (GLCM) is 2D histogram in which (*p*,*q*)th elements is the frequency of event p occurs with *q*. It is a function of distance *S* = 1, angle (at 0 (horizontal), 45° (with the positive diagonal), 90° (vertical) and 135° (negative diagonal) and gray scales *p* and *q*, and calculates how often a pixel with intensity *p*, occurs in relation with another pixel *q* at a certain distance *S* and orientation. In this method, gray-level co-occurrence matrix was initiated and the textural features such as contrast, correlation, energy, homogeneity, entropy and variance were obtained from LL and HL subbands of first four levels of wavelet decomposition [18]. The textural features extracted are listed below:

*Contrast* (*CONT*) Measurement of pixel intensities and its neighbors above image and given by the equation:

2 $$ {\text{CONT}} = \sum\limits_{x = 0}^{m - 1} {\sum\limits_{y = 0}^{n - 1} {(x - y)^{2} f(x,y)} } $$

*Energy* (*ENG*) Energy defines the quantitative amount of repetitive pixel pairs. It is the measurement of affinity in an image, given by equation:

3 $$ {\text{ENG}} = \sqrt {\sum\limits_{p = 0}^{i - 1} {\sum\limits_{q = 0}^{j - 1} {f^{2} (p,q)} } } $$

*Correlation* (*COR*) The measurement of spatial features dependencies between the pixels.

4 $$ {\text{COR}} = \frac{{\sum\nolimits_{p = 0}^{i - 1} {\sum\nolimits_{q = 0}^{j - 1} {(p,q)f(p,q) - M_{{pM_{q} }} } } }}{{\sigma_{p} \sigma_{q} }} $$

*Homogeneity* (*HOM*) Measurement of local uniformity in an image. It is also known as inverse difference moment and contains a single or more range of values to distinguish between textured and non-textured.

5 $$ {\text{HOM}} = \sum\limits_{p = 0}^{i - 1} {\sum\limits_{q = 0}^{j - 1} {\frac{1}{{1 + (p - q)^{2} }}f(p,q)} } $$

*Entropy* (*ENT*) It calculates the designated interference of the textural image. It is given as:

6 $$ {\text{ENT}} = - \sum\limits_{p = 0}^{i - 1} {\sum\limits_{q = 0}^{j - 1} {f(p,q)\log_{2} f(p,q)} } $$

After the textural features extraction, the following features assessment parameter are also required to be obtained for better analysis on brain MRI images.

*Peak signal-to-noise ratio* (*PSNR*) Is a measure used to evaluate the characteristic features of reconstructed image from processed image. It is given as:

7 $$ {\text{PSNR}} = 20\log_{10} \frac{{2^{m} - 1}}{\text{MSE}} $$

Lower the value of mean square error and higher value of peak signal-to-noise ratio indicate better signal-to-noise ratio.

*Mean Square Error* (*MSE*) Measure of fidelity of signal or image. It was used to compare two images by giving quantitative or similarity scores.

8 $$ {\text{MSE}} = \frac{1}{P \times Q}\sum {\sum {\left( {f(i,j) - f^{R} (i,j)} \right)^{2} } } $$

These extracted statistical features were fed into probabilistic neural network (PNN) classifier as an input for training and testing the performance of classifier in the classification of brain tumor images into normal and abnormal.

### Probabilistic neural network (PNN)

In early 1990s, D.F Specht introduced feed-forward neural network named as probabilistic neural network (PNN). It is derived from Bayesian network and statistical algorithm called Kernel Fisher discriminant analysis. It is composed of four nodes or layers: input layer, hidden layer, pattern layer and output layer. PNN formulates the weighted neighbors in the form of neural network [19].

The input layer consists of ‘*P*’ no of neurons that is dependent on categorical variables of various features extracted using gray-level co-occurrence matrix (GLCM). The input node weights were kept 1, and these values were fed into hidden layer. In the pattern layer, Radial basis functions were calculated and were fed in to summation layer. The summation layer adds the weighted values of activation in each class present in hidden layer. The values of summation layer were fed to output layer. The output layer chooses the highest of the probabilities, 1 indicates positive for the target class type and 0 indicates negative for non-targeted class type [20].

## Result and discussion

In this research, we have used two datasets, one was trained dataset collected from Web sites www.diacom.com and the other was test dataset. These datasets were built by experienced radiologists; this includes sample images of five patients with all modalities. The data were collected from digital imaging and communications in medicine dataset. We have considered 650 collected samples from the 25 images of DICOM dataset, of which 18 are infected tumor brain tissues and others normal for the analysis.

Form the survey, the directional features extracted from LL and HL subbands wavelet transform gives the detailed information of different directions with more systematic with characterization changes in biological tissues.

The MRI images were decomposed into five different levels from which the detailed coefficients from LL and HL subbands were selected. These subbands were obtained from wavelet decomposed; the statistical textural features such as energy, correlation, entropy, and homogeneity were extracted using gray-level co-occurrence matrix (GLCM).

The textural features obtained from different levels of wavelet decomposition were taken into consideration and were used as input from training and testing the performance of PNN classifier.

**Fig. 1:**
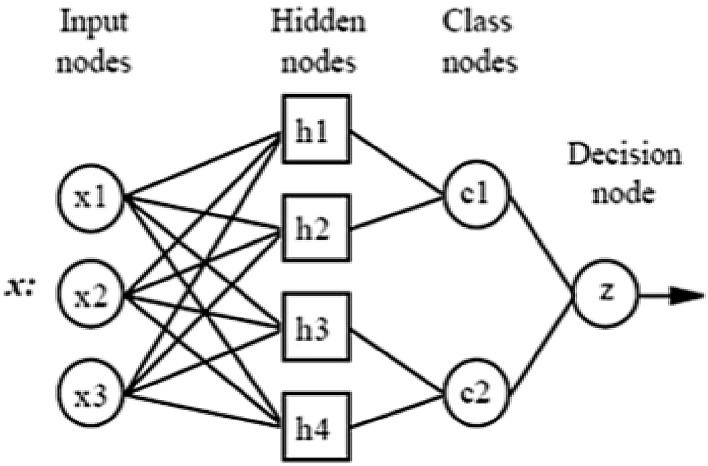
Diagram of probabilistic neural networks

**Fig. 2:**
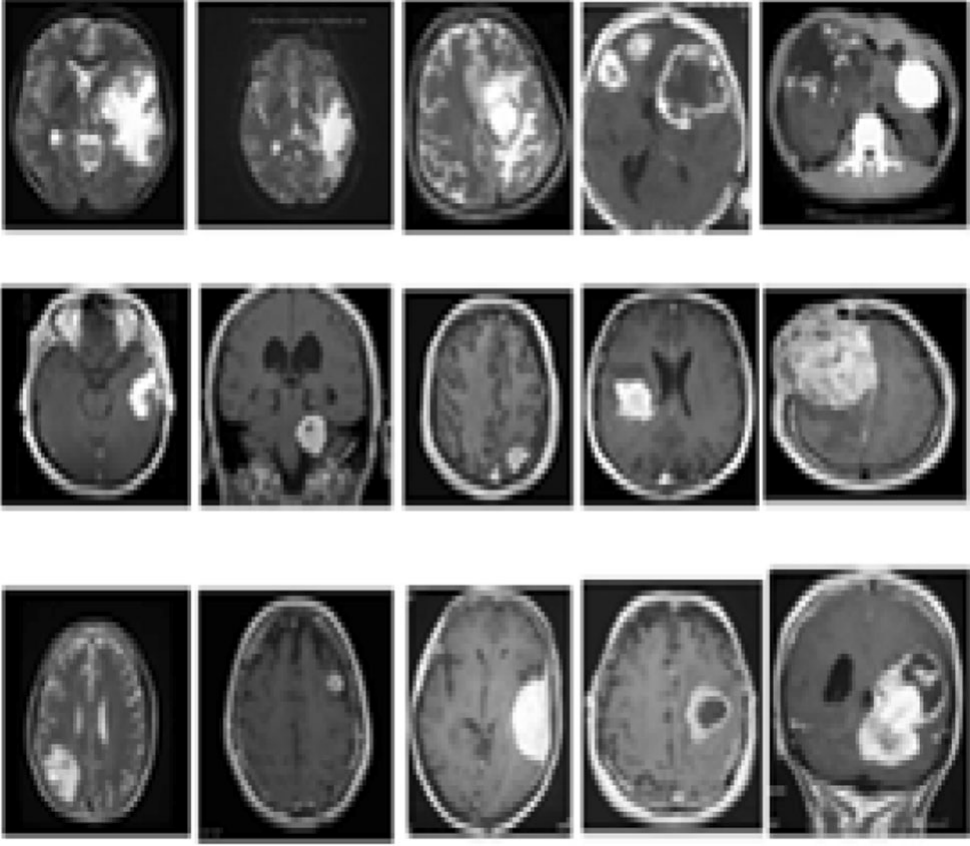
Brain tumor image dataset

The image 1 to image 10 shows different levels of subbands up to 5th level of wavelet decomposition. These extracted features were used as input vectors for training and testing the performance of PNN classifier. Tables 1 and 2 show the statistical textural features such as correlation, contrast, energy, homogeneity and entropy obtained from gray-level co-occurrences matrix formed from different levels of LL and HL subbands of all five levels of trained and tested images (Figs. 1 and 2).

**Table 1:** The statistical features obtained from gray-level co-occurrence matrix (GLCM) of LL and HL subbands of trained images

Images	CON	COR	ENE	HOM	ENT
Image 1	0.0116	0.0710	0.975	0.900	0.337
Image 2	0.0112	0.0206	0.977	0.903	0.332
Image 3	0.0036	0.0381	0.992	0.965	0.339
Image 4	0.0139	0.0067	0.973	0.927	0.395
Image 5	0.0168	0.0259	0.966	0.901	0.337
Image 6	0.0054	0.0027	0.989	0.766	0.272
Image 7	0.0138	0.0069	0.972	0.678	0.275
Image 8	0.0047	0.0288	0.990	0.467	0.337
Image 9	0.0162	0.0081	0.967	0.732	0.272
Image 10	0.0125	0.0477	0.974	0.683	0.337

**Table 2:** The statistical features obtained from gray-level co-occurrence matrix (GLCM) of LL and HL subbands of tested images

Images	CON	COR	ENE	HOM	ENT
Image 1	0.0098	0.0510	0.856	0.930	0.228
Image 2	0.0073	0.0198	0.899	0.870	0.389
Image 3	0.0110	0.0295	0.954	0.910	0.321
Image 4	0.0095	0.0054	0.774	0.882	0.350
Image 5	0.0120	0.0243	0.832	0.891	0.302
Image 6	0.0043	0.0034	0.820	0.745	0.253
Image 7	0.0100	0.0056	0.854	0.798	0.265
Image 8	0.0030	0.0266	0.860	0.950	0.330
Image 9	0.0130	0.0071	0.789	0.947	0.232
Image 10	0.0108	0.0450	0.893	0.864	0.330

The performance analysis of segmented images with the calculation of area is tabulated in Table 3. A lower value of MSE and a higher value of PSNR indicate better signal-to-noise ratio in the extracted image.

**Table 3:** The performance evaluation and area calculation of tumor extracted region of trained images

Images	PSNR	MSE	Area of image in pixel	Area of tumor region
Image 1	14.011	6.121	39,240	7698
Image 2	13.82	3.116	67,824	9874
Image 3	14.12	8.068	50,508	7423
Image 4	13.86	4.77	50,388	9056
Image 5	13.79	5.84	24,964	4564
Image 6	13.82	7.79	50,429	3698
Image 7	13.99	6.92	50,298	5879
Image 8	14.004	7.35	35,040	13,923
Image 9	14.066	6.215	50,544	6534
Image 10	14.03	6.172	16,384	4497

From the observation, the contrast of trained MR images obtained was found to be more when compared to tested MR images, whereas the homogeneity of trained MR images was found to be less when compared to tested MR images. Similarly, the entropy and energy are found more in trained MR images when compared to tested MR images. With this proposed methodology and with the help of statistical textural features (contrast, correlation, energy, homogeneity and entropy) procured from LL and HL subbands classified the brain tumor images into normal and abnormal. The differences in statistical textural feature values of trained and tested brain tumors were found to be very useful in manipulating the performance of the PNN classifier in training and testing.

**Fig. 3:**
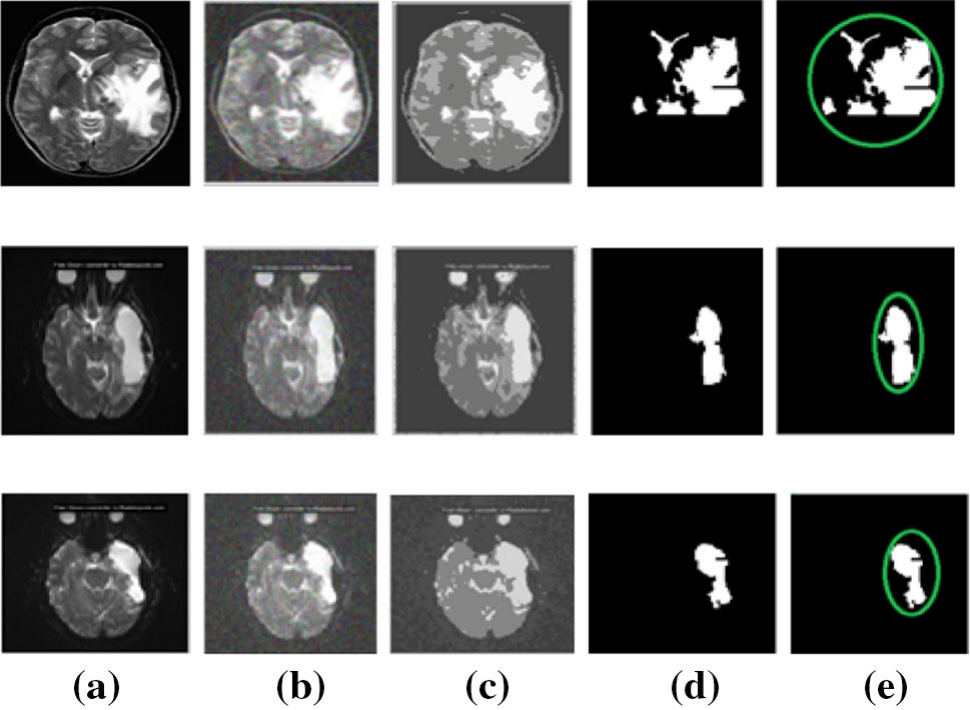
Observational results of an image **a** original images, **b** preprocessed images, **c** region segmentation tumor image, **d** extracted tumor images, **e** area of extracted tumor region

The observation results are shown in Fig. 3 representing original images (a) column wise, (b) preprocessed images obtained by filtering of noise, (c) region-based segmentation images, (d) extracted tumor-affected region from segmented images, area of the tumor-affected region.

## Performance analysis

**Fig. 4:**
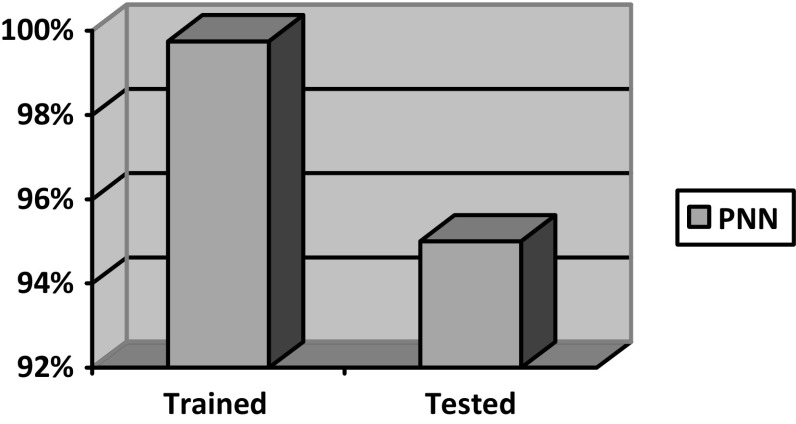
Comparison of trained and tested dataset classification using probabilistic neural networks

The trained dataset images for which the features extracted were trained using probabilistic neural network (PNN) classifier for the classification purpose, whereas the test dataset was not trained using PNN classifier, only the statistical and textural features were extracted. The accuracy of trained and tested image was compared based on the classification of normal and abnormal tumor tissues. Figure 4 shows the accuracy results in classification of normal and abnormal tumor tissues.

Accuracy or correct rate of classification is the efficiency of appropriate classification to the total number of classification tests [19]. This process of brain tumor classification has been performed on various normal and abnormal MR images, and the accuracy of the PNN classifier is manipulated, using the equation given below:

9 $$ {\text{Accuracy}}\left( \% \right) = \frac{{{\text{Correct}}\;{\text{cases}}}}{{{\text{Total}}\;{\text{number}}}} \times \, 100 $$

## Conclusion and future scope

In this research, we have used brain MR images, segmented into normal brain tissue (unaffected) and abnormal tumor tissue (infected). To remove a noise and smoothen the image, preprocessing is used which also results in the improvement of signal-to-noise ratio. Next, we have used discrete wavelet transform that decomposes the images and textural features were extracted from gray-level co-occurrence matrix (GLCM) followed by morphological operation. Probabilistic neural network (PNN) classifier is used for the classification of tumors from brain MRI images.

From the observation results, it can be clearly expressed that the detection of brain tumor is fast and accurate when compared to the manual detection carried out by clinical experts. The performance factors evaluated also shows that it gives better outcome by improving PSNR and MSE parameters.

The proposed methodology results in accurate and speedy detection of tumor in brain along with identification of precise location of the tumor.

In identification and classification into normal and abnormal tumors from brain MR images, accuracy of nearly 100% was achieved for trained dataset because the statistical textural features were extracted from LL and HL subbands wavelet decomposition and 95% was achieved for tested dataset. With the above results, we conclude that our proposed method clearly distinguishes the tumor into normal and abnormal which helps in taking clear diagnosis decisions by clinical experts.

In the future work, different classifiers can be used to increase the accuracy combining more efficient segmentation and feature extraction techniques with real- and clinical-based cases by using large dataset covering different scenarios.
